# Morphologically-Directed Raman Spectroscopy as an Analytical Method for Subvisible Particle Characterization in Therapeutic Protein Product Quality

**DOI:** 10.1038/s41598-023-45720-0

**Published:** 2023-11-22

**Authors:** Minkyung Kim, Youlong Ma, Charudharshini Srinivasan, Thomas O’Connor, Srivalli N. Telikepalli, Dean C. Ripple, Scott Lute, Ashwinkumar Bhirde

**Affiliations:** 1 Division of Biotechnology Research and Review II, Office of Biotechnology Products, Office of Pharmaceutical Quality, Center for Drug Evaluation and Research, U.S. Food and Drug Administration, Silver Spring, MD USA; 2 Division of Product Quality Research, Office of Testing and Research, Office of Pharmaceutical Quality, Center for Drug Evaluation Research, U.S. Food and Drug Administration, Silver Spring, MD USA; 3 Biomolecular Measurement Division, National Institute of Standards and Technology, Gaithersburg, MD USA

**Keywords:** Biotechnology, Cancer, Analytical chemistry, Materials chemistry, Characterization and analytical techniques, Imaging techniques, Microscopy, Optical spectroscopy

## Abstract

Subvisible particles (SVPs) are a critical quality attribute of injectable therapeutic proteins (TPs) that needs to be controlled due to potential risks associated with drug product quality. The current compendial methods routinely used to analyze SVPs for lot release provide information on particle size and count. However, chemical identification of individual particles is also important to address root-cause analysis. Herein, we introduce Morphologically-Directed Raman Spectroscopy (MDRS) for SVP characterization of TPs. The following particles were used for method development: (1) polystyrene microspheres, a traditional standard used in industry; (2) photolithographic (SU-8); and (3) ethylene tetrafluoroethylene (ETFE) particles, candidate reference materials developed by NIST. In our study, MDRS rendered high-resolution images for the ETFE particles (> 90%) ranging from 19 to 100 μm in size, covering most of SVP range, and generated comparable morphology data to flow imaging microscopy. Our method was applied to characterize particles formed in stressed TPs and was able to chemically identify individual particles using Raman spectroscopy. MDRS was able to compare morphology and transparency properties of proteinaceous particles with reference materials. The data suggests MDRS may complement the current TPs SVP analysis system and product quality characterization workflow throughout development and commercial lifecycle.

## Introduction

There has been a breakthrough of therapeutic proteins (TPs) over the past 30 years in human medicine^1^. TPs play a crucial role in almost every field of human medicine, and their growth is expected to increase^2^. Considering the recent advance of TPs, it is important to better characterize critical quality attributes to ensure the desired product quality throughout the drug product life cycle. Particulate matter in TPs has been considered a key challenge in quality control having potential risks of triggering immunogenicity^3–5^. Particles can be distinguished as extrinsic, intrinsic, and inherent by source. By size, they are categorized into visible (> 100 µm), subvisible (1–100 µm), submicron (100–1000 nm), and nanometer (< 100 nm) particles. Particles in the micrometers size range are classified into subvisible or visible particles, but there is no distinct size cut-off based on the actual visibility of the particles^6^. The probability of detection depends on the nature of particles; many factors such as the particle’s optical properties, morphology, size, etc. can impact instrument detection of the particle^7^.

To control and monitor subvisible particles (SVPs), the current USP guidelines suggest that particulates ≥ 10 μm in size are controlled at or below 6000 particles per container and particles ≥ 25 μm are limited to at or below 600 particles/container^8^. For particle detection, light obscuration (LO) and membrane microscopy (MM) methods have been used as the current compendial methods, and flow imaging microscopy (FIM) has been recommended as the orthogonal method based on USP < 1787 > and < 1788 > . Based on USP < 787 > , LO is the most widely used compendial method which provides particle numbers in a given size range but it has been reported that LO typically underestimates the counts and sizes of proteinaceous particles as these values are significantly influenced by the optical property of a sample^9–11^. MM systems can render particle images and morphological data along with particle counts, however, chemical identification of the particles is not possible^12,13^. Although FIM systems have rapid throughput and a sensitive limit of quantitation, definitive identification of particle type is often not possible, and methods for chemical identification of particles are needed. Electron microscopy (EM) methods listed in USP < 1787 > such as scanning EM (SEM), transmission EM (TEM), and scanning transmission EM (STEM) are able to identify particles using energy dispersive spectrometry (EDS). Although EDS offers elemental analysis^14^, it requires a complicated sample preparation process that might change the sample native state.

In SVP analysis, polystyrene (PS) microspheres are commonly-used particle standards during method validation in the industry and they have monodispersed spherical shape and size with high optical contrast, whereas particles observed in TPs are polydisperse with mixed shape and size, and have low optical contrast^15^. For these reasons, the National Institute of Standards and Technology (NIST) has been developing proteinaceous particle surrogates as candidate reference materials such as epoxy-based photo-resist SU-8 and ethylene tetrafluoroethylene (ETFE) particles to help characterization of SVPs in injectable TPs^16,17^. The SU-8 and ETFE particles are synthetic polymers, and they have much better stabilities with similar optical contrast to proteinaceous particles. The SU-8 particles are monodispersed with uniformed morphology, and therefore can be used to compare different particle analysis algorithms used by various instruments during particle morphology analysis. The ETFE particles are polydisperse and translucent having irregular morphology and low optical contrast much like proteinaceous particles. Considering the nature of these particles, they have potential to be better alternatives to traditional standards such as PS microspheres and barium sulfate salts^17^.

Morphologically-Directed Raman Spectroscopy (MDRS) has been utilized to determine the particle size distribution (PSD) of nasal suspensions for demonstrating bioequivalence^18^. In this study, we introduce an analytical method using MDRS for proteinaceous SVP identification and characterization. This method was developed using PS microspheres, SU-8 and ETFE particle suspensions and applied to characterization of particles formed in stressed monoclonal antibody (mAb) samples, which can be used for TPs quality control. Different aspects of MDRS and FIM in particle characterization were discussed with respect to image quality, morphology evaluation, particle size distribution analysis and particle identification. Unlike the post-filtration analysis of MM, MDRS can evaluate the SVP in their native state, avoiding distortion of soft proteinaceous particles. In addition, previous high-throughput particle analysis studies have focused on particle quantitation and conducted particle identification solely depending on their images^9–13^. Our findings show that along with detailed morphological information, MDRS provides chemical identification of individual particles, which is more definitive approach. This highlights its prospect as another orthogonal method in SVP characterization during drug development stage as well as root cause analysis in product quality characterization of TPs.

## Results

### Image analysis

**Figure 1:**
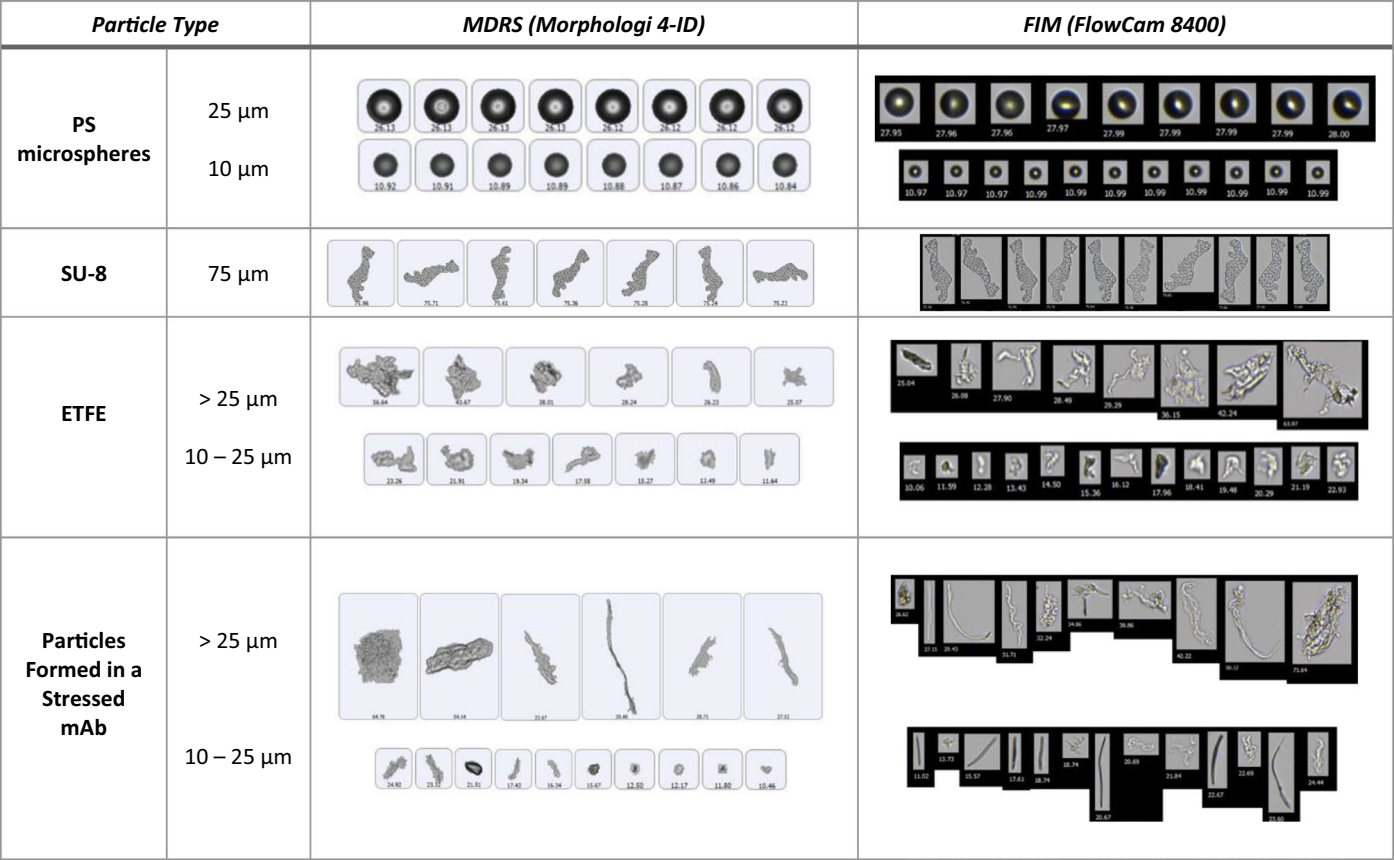
Representative images of PS microspheres, SU-8 particles, ETFE particles, and particles formed in a stressed mAb sample captured by MDRS and FIM.

Particle morphology evaluation is very dependent on image quality. In this study, two particle imaging systems, namely, MDRS (Morphologi 4-ID from Malvern Panalytical) and FIM (FlowCam 8400 from Yokogawa Fluid Imaging Technologies) were used for SVP analysis. In TPs quality control, FIM has been widely used in industry for SVP analysis after LO. This experiment was designed to compare the imaging capability of MDRS system for SVP analysis with the current orthogonal method FIM system. Three particle standards PS microspheres (a traditional reference material), SU-8, and ETFE (candidate reference materials) were used for comparison, and particles ≥ 10 μm were investigated based on USP < 788 > . Overall, as shown in Fig. 1, both MDRS and FIM systems provided high-resolution images for all monodisperse particle standards, PS microspheres and SU-8. When imaging the polydisperse ETFE particles, the FIM system produced sharp and high-resolution images for most of the particles (> 90%) in the range from 10 to 100 μm.

MDRS showed greater than 90% probability in capturing high-resolution images for the polydisperse ETFE particles ranging from 19 to 100 μm, and the resolution decreased as the particle size decreased, as shown in Table 1. When capturing particles in the range of 16–17 μm, the probability decreased to 70%. MDRS occasionally fragments large particles into smaller ones, thereby lowering the quality of the high-resolution images of the smaller particles in the measurement, as shown in Figure S1. MDRS utilizes a static imaging system, and this enabled us to manually assess particle image resolutions. Particles remain in the sample holder after each measurement; this allows users to go back to individual particles. In this manner, machine-determined outlines were compared side-by-side with brightfield optical microscopy inspection of MDRS and the particle images with clear outlines with no fragmentation were considered high-resolution images (Table 1).

**Table 1:** The imaging performance of Morphologi 4-ID depending on size of particles. The processed images were captured in each measurement and compared with the original particles of interest shown under microscope.

Particle size (μm)				High quality image (%)			
≥ 19 μm				> 90			
18–19				78.6			
17–18				76.9			
16–17				70.0			

Particle ID	Diameter (μm)	Particle of interest	Processed image	Particle ID	Diameter (μm)	Particle of interest	Processed image
1689	56.6			3399	17.4		
636	34.4			1176	16.1		
2523	25.1			1958	15.3		
3031	20.1			2208	14.5		
3298	18.2			1647	13.1		

### Size and shape analysis of monodisperse particle standards (PS microspheres and SU-8 candidate materials)

**Figure 2:**
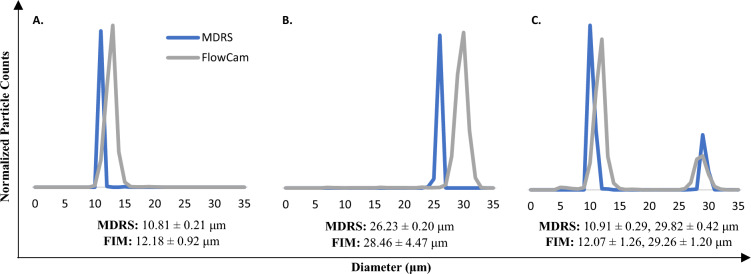
Size measurement analysis of PS microspheres standards using MDRS and FIM. The 10 μm (**A**) and 25 μm (**B**) microsphere standards were measured separately and, in a mixture (**C**). Particle sizes are obtained from 3 separate measurements (n = 3) reported as the mean ± standard deviation. Segmentation threshold (dark/light) were set for 12/12 in the FIM measurements.

The size and shape analysis capabilities of MDRS and FIM systems were evaluated for comparative study using monodisperse particles. Overall, the MDRS provided comparable results to the FIM system (Fig. 2), when using the same thresholds for proteinaceous particle analysis. First, 10 μm and 25 μm microspheres were used for this study. Considering the specification values of PS microsphere standards (10.02 ± 0.06 μm and 25.52 ± 0.34 μm), the FIM slightly overestimated the sizes of microspheres in the measurements of all single particle samples (12.18 ± 0.92 μm for 10 μm microspheres and 28.46 ± 4.47 μm for 25 μm microspheres) and a mixed particle sample (12.07 ± 1.26 for 10 μm microspheres and 29.26 ± 1.20 μm for 25 μm microspheres), even though it rendered focused and well-resolved images. Overall, the size measurement results (10.81 ± 0.21 μm for 10 μm microspheres in a single particle sample, 10.91 ± 0.29 μm for 10 μm microspheres in a mixture, and 26.23 ± 0.20 μm for 25 μm microspheres in a single particle sample) provided by MDRS were closer to the specifications. The MDRS measurement of 25 μm microspheres in the mixture (29.82 ± 0.42 μm) showed similar deviation to FIM measurement. In a different threshold setting, FIM offered better results of average particle sizes in the measurements of single particle samples (10.81 ± 2.26 μm for 10 μm microspheres and 27.70 ± 2.78 μm for 25 μm microsphere) and a mixed particle sample (10.87 ± 1.31 μm for 10 μm and 27.91 ± 1.41 μm for 25 μm microspheres). Despite closer values to the specifications, much broader particle size distributions were observed, as shown in Figure S2.

**Figure 3:**
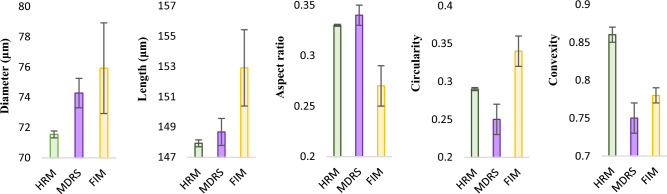
Morphological data of SU-8 particles calculated by HRM (high-resolution microscopy), MDRS and FIM. The maximum distance between any two parallel tangents on a particle, also known as the maximum Feret’s diameter, was presented as length. Morphological values were obtained from 3 separate measurements (n = 3) reported as the mean ± standard deviation. Diameters measured by HRM, MDRS, and FIM were 71.55 ± 0.23, 74.28 ± 0.97, and 75.92 ± 2.99, respectively. Lengths measured by HRM, MDRS, and FIM were 147.92 ± 0.23, 148.67 ± 0.89, and 152.9 ± 2.52, respectively. Aspect ratios measured by HRM, MDRS, and FIM were 0.33 ± 0.001, 0.34 ± 0.01, and 0.27 ± 0.02, respectively. Circularity measured by HRM, MDRS, and FIM were 0.29 ± 0.002, 0.25 ± 0.02, and 0.34 ± 0.02, respectively. Convexity measured by HRM, MDRS, and FIM were 0.86 ± 0.01, 0.75 ± 0.02, and 0.78 ± 0.01, respectively.

The SU-8 particles consist of monodisperse particles with a well-defined shape. Given that different instruments employ different algorithms when calculating morphological data, this candidate reference material was used to assess the accuracy and compare size and shape measurement in two systems. Morphological data such as diameter, length, aspect ratio, circularity, and convexity values were calculated by MDRS and FIM, and these values were compared with the values directly obtained from optical microscopy measurements, as shown in Fig. 3. As seen in the evaluation of size measurements of PS microspheres, most of morphology values measured by MDRS were precise with a smaller standard deviation. In the size evaluation, diameter (74.28 ± 0.97 μm) and length (148.67 ± 0.89 μm) measured by MDRS were closer to the results from optical microscopy (71.55 ± 0.23 μm for diameter and 147.92 ± 0.23 μm for length) than diameter (75.92 ± 2.99 μm) and length (152.9 ± 2.52 μm) measured by FIM. The shape evaluation illustrated a similar trend as the size evaluation. Aspect ratio (0.34 ± 0.01) and circularity (0.25 ± 0.02) calculated by MDRS were closer to the optical microscopy values (0.33 ± 0.001 for aspect ratio and 0.29 ± 0.002 for circularity) compared to aspect ratio (0.27 ± 0.02) and circularity (0.34 ± 0.02) values calculated by FIM. Convexity was the only morphology factor that FIM (0.78 ± 0.01) calculated closer value to optical microscopy (0.86 ± 0.01) compared to MDRS (0.75 ± 0.02). P values for all the morphology factors were less than 0.001 regardless of methods indicating statistical significances. In summary, our data indicated that MDRS offered comparable size and shape information to morphological values analyzed by optical microscopy in most of measurements.

### Size and shape analysis of polydisperse ETFE particles

**Figure 4:**
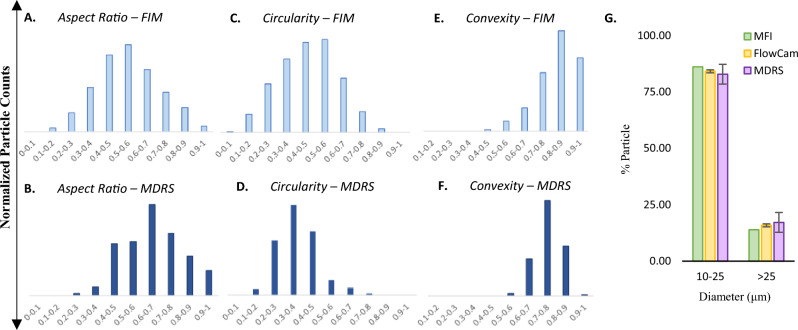
(**A**)–(**F**) Morphological data histograms and (**G**) particle size distributions of ETFE particles measured by different methods. FlowCam and MDRS systems were used to measure (**A**) & (**B**) aspect ratio, (**C**) & (**D**) circularity, and (**E**) & (**F**) convexity of the ETFE particles. (**G**) The particle size distribution was measured by MDRS (purple) and compared with the two FIM systems, MFI (green) and FlowCam (yellow).

Next, the size and shape analysis capabilities of MDRS and FIM systems were evaluated using polydisperse ETFE particles. First, in shape analysis, the MDRS results were comparable with the FIM results. Given that high-resolution images were obtained with > 90% probability by MDRS for particles > 19 μm, morphology data of the particles in this size range were used to plot the histograms in comparison of shape analysis performance, as shown in Fig. 4A–F. Morphology parameters such as aspect ratio, circularity, and convexity are defined and explained in Method section. Aspect ratio, circularity, and convexity values calculated by FIM were 0.56 ± 0.16, 0.47 ± 0.15, and 0.83 ± 0.11, respectively. MDRS determined aspect ratio, circularity, and convexity values as 0.65 ± 0.15, 0.35 ± 0.10, and 0.74 ± 0.07, respectively. Overall, both systems presented similar distributions in morphology values, but for all the morphology factors, *p* values between MDRS and FIM methods were less than 0.001 demonstrating statistical significances.

The particle size data obtained by MDRS (Morphologi 4-ID) were compared with two different FIM systems, MFI (Micro-Flow Imaging DPA4200) and FlowCam (FlowCam8400), as shown in Fig. 4G. MFI and FlowCam systems provided comparable size data with each other as reported^19^. FIM systems count the number of particles in a specified volume, which remains relatively constant for each measurement with narrow error bars. However, the particle concentrations measured by MDRS varies in each measurement because the concentration quantitation was largely affected by particle dispersion states. For this reason, MDRS has been used to provide percentage-based analysis such as % particle size distribution and % particle composition^20–22^. Hence, % particle size distribution was investigated using ETFE for a comparative study between FIM and MDRS. MFI, FlowCam, and MDRS calculated 86.08%, 84.13 ± 0.66%, and 82.84 ± 4.38% for particles ranging from 10 to 25 μm, respectively. For particles ≥ 25 μm, 13.92%, 15.87 ± 0.66%, and 17.17 ± 4.39% were measured by MFI, FlowCam, and MDRS, respectively. These systems reported similar particle size distribution results, showing less than 3.25% differences for both size ranges. Even though MDRS shows the biggest standard deviation, the size analysis performance of MDRS were comparable to the other two systems given that approximately 4.4% standard deviation is not considered as a statistically significant variation.

### Particle identification using MDRS

**Figure 5:**
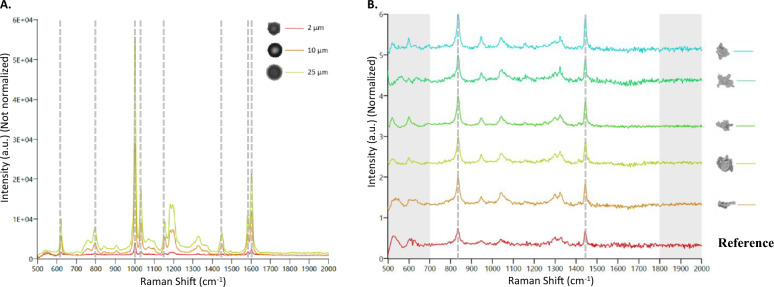
(**A**) The overlaid Raman spectra of PS microspheres with different sizes. 2 μm, 10 μm, and 25 μm microspheres were examined and the peak intensities were not normalized for the direct comparison of the intensity change. Characteristic peaks found in PS microspheres were highlighted with gray dashed lines at 621 cm^−1^, 795 cm^−1^, 1001 cm^−1^, 1031 cm^−1^, 1155 cm^−1^, 1450 cm^−1^, 1583 cm^−1^and 1602 cm^−1^. (**B**) Representative Raman spectra of ETFE particles measured by MDRS. ETFE particles ≥ 25 μm were investigated and their intensities were normalized. The region of 700–1800 cm^−1^ in each spectrum was compared with ETFE reference spectrum (red) and their correlation scores were calculated based on their peak similarity. Characteristic peaks found in an ETFE polymer were highlighted with gray dashed lines at 835 cm^−1^, and 1444 cm^−1^.

Compared to other SVP imaging methods, the biggest advantage of MDRS is a direct chemical identification of each particle using Raman spectroscopy^23–25^. In this study, PS microspheres and ETFE particles were used to assess its particle identification capability. Chemical correlation scores of their Raman spectra to reference spectra were calculated and the scores between 0 (the least identical to the reference spectrum) and 1 (the most identical to the reference spectrum) were assigned to each particle. A mixture of PS microspheres in three different sizes (2 μm, 10 μm, and 25 μm) were used and Raman spectra of 30 out of 622 particles (10 particles for each size) in a batch were measured individually using a 30 s exposure time, as described in the "Methods" section. PS microspheres are known to have eight characteristic peaks in its Raman spectrum such as aromatic ring deformation at 621 cm^−1^, C–H out-of-plane deformation at 795 cm^−1^, aromatic ring breathing mode at 1000 cm^−1^, C–H in-plane deformation at 1031 cm^−1^, C–C stretch at 1155 cm^−1^, CH_2_ scissoring at 1450 cm^−1^, C=C stretch at 1583 cm^−1^, and aromatic ring-skeletal stretch at 1602 cm^−1^^26,27^. As shown in Fig. 5A, Raman spectra of a mixture of 2 μm, 10 μm, and 25 μm PS microspheres were overlaid, indicating that the signal intensity decreased as the sizes of microspheres decreased. The laser size of an MDRS instrument is 3 μm, and therefore it attenuates the signal-to-noise ratio (S/N) of particles having smaller than 3 μm because of background interference^18^. Despite a relatively low S/N for 2 μm PS microspheres peaks compared to the larger microspheres, the average chemical correlation scores of 2 μm, 10 μm, and 25 μm microspheres were calculated as 0.96, 1.00, and 1.00, respectively, enabling successful particle identification.

Raman spectra of all the ETFE particles ≥ 25 μm (90 out of 90) and 100 out of 1044 particles in the range of 5–25 μm were manually acquired. ETFE is a fluorine-based polymer and the ETFE Raman spectrum is dominated by two sharp peaks at 835 cm^−1^ for CF_2_ stretch and 1444 cm^−1^ for CH_2_ bending^28^. The representative Raman spectra of ETFE particles ≥ 25 μm is shown in Fig. 5B and the average chemical correlation score was calculated as 0.90 out of 1, closely matching the reference ETFE spectrum. The average chemical correlation scores of the particles in range of 10–25 μm and 5–10 μm were calculated to be 0.83 and 0.65, respectively. These results indicate that as the sizes of particles decrease, the average correlation score decreases also due to lower S/N, which made it more challenging for the software to differentiate the particle of interest. The representative Raman spectra of each particle group can be found in Figure S3.

### Characterization of proteinaceous particles formed in stressed mAb samples using MDRS

**Figure 6:**
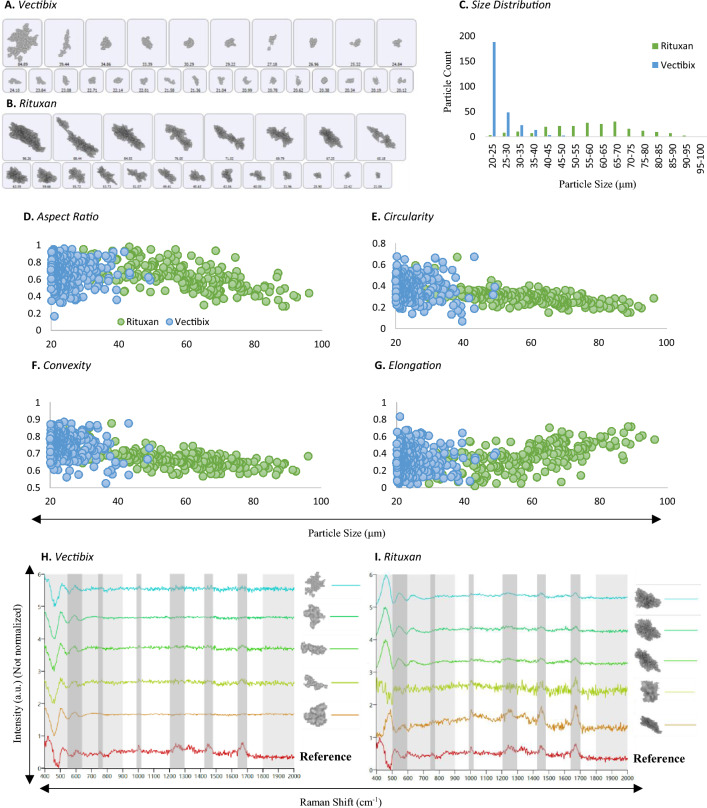
(**A**) (**B**) Representative particle images, (**C**) particle size distributions, (**D**)–(**G**) morphological data histograms, (**H**) (**I**) representative Raman spectra of proteinaceous particles in stressed mAb samples, Vectibix (blue) and Rituxan (green), measured by MDRS system. Morphological data such as (**D**) aspect ratio, (**E**) circularity, (**F**) convexity, and (**G**) elongation values of the two proteinaceous particles were compared. In Raman spectra, characteristic peaks of proteinaceous particles were highlighted in gray regions at 500–550 cm^−1^, 770 cm^−1^, 1000 cm^−1^, 1230 cm^−1^, 1400 cm^−1^, and 1670–1690 cm^−1^. The region of 900–1800 cm^−1^ in each spectrum was compared with protein aggregate reference spectrum (red) and their correlation scores were calculated based on the peak similarity.

MDRS particle analysis method was applied to characterize particles in stressed TPs. Two commercially available mAb drug products—Vectibix and Rituxan—were subjected to stress condition (70 °C and 300 rpm) to artificially generate particles as described in the "Methods" section. Considering image resolution, particles > 20 μm were investigated among all the particles formed in the mAb samples. Particle size distributions and morphology parameters such as aspect ratio, circularity, convexity, and elongation were examined. Another shape parameter, elongation, was added to take a detailed look at fibril-shaped particle formation. In addition to the morphology analysis, each particle was chemically identified using Raman spectroscopy. The representative images of individual particles were shown in Fig. 6A for Vectibix and Fig. 6B for Rituxan. The Vectibix sample yielded gray and transparent particles compared to dark particles formed in the Rituxan sample. The size distribution histogram shown in Fig. 6C indicated that most of particles in the Vectibix sample were smaller than 40 μm. In contrast, particles in Rituxan sample had various sizes across the SVP range from 20 to 100 μm, even though they were exposed to the same stress conditions.

Particle morphology parameters were studied and shown in Fig. 6D–G and the average values were summarized in Table 2. Our data indicated that the average morphology values of the particles formed in the Vectibix sample did not change much as the particle size grew. The average aspect ratio value slightly increased from 0.67 to 0.69 and the other values slightly decreased from 0.39 to 0.37 for circularity, from 0.75 to 0.70 for convexity, and from 0.33 to 0.31 for elongation. In contrast, the average morphology values of the particles formed in Rituxan sample showed different trends. As particle growth, elongation was the only value increased from 0.27 to 0.55 and other values decreased from 0.74 to 0.45 for aspect ratio, from 0.4 to 0.21 for circularity, and from 0.74 to 0.62 for convexity. Compared to the differences in morphology values of Vectibix particles, significant increase or decrease were observed in Rituxan particles.

**Table 2:** The average morphological values of the particles formed in Vectibix and Rituxan samples as particle growth.

Diameter	20–40 µm		40–60 µm		60–80 µm		80–100 µm	
Average	Vectibix	Rituxan	Vectibix	Rituxan	Vectibix	Rituxan	Vectibix	Rituxan
Aspect ratio	0.67	0.74	0.69	0.71	–	0.56	–	0.45
Circularity	0.39	0.40	0.37	0.30	–	0.27	–	0.21
Convexity	0.75	0.74	0.70	0.67	–	0.65	–	0.62
Elongation	0.33	0.27	0.31	0.30	–	0.44	–	0.55

The Raman spectra of particles from stressed mAb samples are presented in Fig. 6H for Vectibix and Fig. 6I for Rituxan. Among particles > 25 μm, 57 out of 531 particles in Rituxan sample and 26 out of 91 particles in Vectibix sample were randomly selected and their Raman spectra were manually acquired. Proteins misfold or unfold when they are exposed to various stress conditions such as acidic/basic pH, heat, and agitation, resulting in protein aggregation. Consequently, peak shift, broadening and/or peak intensity change can be observed in certain peaks of the proteinaceous particle Raman spectrum, but in general, they have similar Raman spectra to the native protein. The characteristic peaks of mAbs are highlighted in gray regions at 500–550 cm^−1^ for disulfide bonds, at 770 cm^−1^ for tryptophan, at 1000 cm^−1^ for phenylalanine, at 1230 cm^−1^ for amide III bonds, at 1400 cm^−1^ for asymmetric CH_2_, and at 1670–1690 cm^−1^ for amide I bonds. Since the tryptophan peak at 760 cm^−1^ is usually weak, the region from 900 to 1800 cm^−1^ was investigated to calculate chemical correlation scores. Among all the proteinaceous particle spectra, as a protein aggregate reference, selected was the spectrum having the strongest peaks for phenylalanine, amide III bonds, asymmetric CH_2_, and amide I bonds. With respect to the reference spectrum, the average chemical correlation scores of particles in Vectibix and Rituxan samples were calculated as 0.35 and 0.66, respectively. The average chemical correlation scores of Rituxan proteinaceous particles to ETFE and PS microsphere reference spectra were calculated as 0.26 and 0.09, respectively. The average chemical correlation scores of Vectibix proteinaceous particles to ETFE and PS microsphere reference spectra were calculated as 0.16 and 0.05, respectively. Rituxan proteinaceous particles showed higher chemical correlation score regarding protein aggregate reference because they were larger and thicker, resulting in higher S/N, compared to the Vectibix proteinaceous particles. The correlation scores of Rituxan proteinaceous particles indicated a meaningful difference between protein aggregate and other standards such as ETFE and PS microsphere. In contrast, the correlation scores of Vectibix proteinaceous particles demonstrated insignificant difference between protein aggregate and other standards due to low S/N. The chemical correlation scores generally decrease, as particle size decreases. In case of Rituxan proteinaceous particles, among all the proteinaceous particles bigger than 40 μm, the lowest chemical correlation score was 0.6 and the average score was 0.78 out of 1. As particle sizes reached below 40 μm, their chemical correlation scores started to decrease rapidly, as shown in Figure S4.

### Suitability of ETFE particles as a proteinaceous particle surrogate

**Figure 7:**
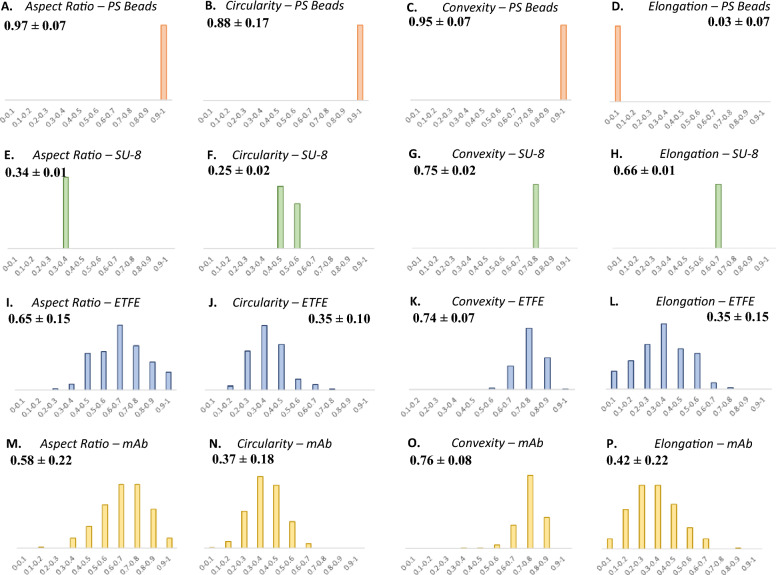
(**A**)–(**D**) Morphological data histograms of PS microspheres (orange), (**E**)–(**H**) SU-8 particles (green), (**I**)–(**L**) ETFE particles (blue), and (**M**)–(**P**) proteinaceous particles (yellow) in a stressed mAb (Vectibix) sample measured by MDRS. Aspect ratio, circularity, convexity, and elongation histograms were compared.

Morphology parameters of three particle reference materials such as PS microspheres, SU-8, and ETFE were measured by MDRS and compared with those of proteinaceous particles formed in the Vectibix sample. Considering image resolutions, data of the monodisperse particles (PS microspheres and SU-8) in all size ranges and polydisperse particles (ETFE and mAb) > 20 um were used to calculate these values. The histograms of aspect ratio, circularity, convexity, and elongation values are shown in Fig. 7. The aspect ratio values were calculated to be 0.97 ± 0.07 for PS microspheres, 0.34 ± 0.01 for SU-8, 0.65 ± 0.15 for ETFE, and 0.58 ± 0.22 for mAb proteinaceous particle. The circularity values were calculated as 0.88 ± 0.17 for PS microspheres, 0.25 ± 0.02 for SU-8, 0.35 ± 0.10 for ETFE, and 0.37 ± 0.18 for mAb proteinaceous particle. The convexity values were calculated as 0.95 ± 0.07 for PS microspheres, 0.75 ± 0.02 for SU-8, 0.74 ± 0.07 for ETFE, and 0.76 ± 0.08 for mAb proteinaceous particle. The elongation values were calculated as 0.93 ± 0.07 for PS microspheres, 0.66 ± 0.01 for SU-8, 0.35 ± 0.15 for ETFE, and 0.42 ± 0.22 for mAb proteinaceous particle.

Transparency is an important particle property along with particle morphology. To investigate differences in transparency between particle reference materials and proteinaceous particles, mean intensity values of PS microspheres, SU-8, and ETFE were measured by MDRS and compared with those of proteinaceous particles formed in the Rituxan and Vectibix samples, as shown in Figure S5. A particle with high mean intensity is considered a more transparent particle. Our data reported that PS microspheres showed a much lower value of 58.97 ± 8.15, while in contrast SU-8 (143.45 ± 3.87) and ETFE (156.10 ± 6.86) particles showed similar values to proteinaceous particles (135.42 ± 6.39 for Rituxan and 164.32 ± 4.41 for Vectibix). In all aspects, ETFE candidate reference material demonstrated the closest values and PS microspheres exhibited the farthest values to the values obtained from the proteinaceous particles. In morphology analysis, the ETFE histogram distributions resembled those of the proteinaceous particles the most, in that they showed wide distributions of morphology values. In contrast, the histograms of the other particle reference materials such as PS microspheres and SU-8 showed very narrow value ranges. Transparency analysis reported a similar result demonstrating that ETFE particles are as transparent as proteinaceous particles.

## Discussion

SVP matter is a critical quality attribute of TPs, and it is necessary to apply advanced technologies to the characterization of the SVPs. The current compendial method LO and its orthogonal methods such as FIM and MM have been routinely utilized to analyze SVPs in TPs for lot release based on USP guidelines. However, these methods cannot distinguish particle types, even though particle identification is important to understand particle origins, given that potential SVP contaminants in TPs can range from glass, silicone/silica, fiber, polymers, rubber, metal, dust, to hair etc^29^. Herein, we developed a new SVP analysis method using MDRS for product quality characterization of TPs. For image analysis of monodisperse particles such as PS microspheres (10 μm and 25 μm) and SU-8 (75 μm), both MDRS and FlowCam particle imaging systems provided well-resolved images**.** However, for image analysis of the polydisperse ETFE particles, MDRS rendered high-resolution images for the SVPs ≥ 19 μm, while FlowCam produced high-resolution images of the particles across the subvisible size range from 2 to 80 μm. Our results demonstrated that the quality of particle images produced by MDRS were impacted by two factors: the particle shape and size range. First, in MDRS measurements, particle image resolution depends on particle shape. Our data suggested that high-resolution images were provided for 10 μm PS microspheres but low-resolution images were generated from ETFE particles in the same size. This may be because the PS microsphere has a monodispersed spherical shape with a smooth surface and the ETFE particle has an irregular shape with an uneven surface. Processing particle images with uneven surfaces could be a challenge in MDRS analysis.

Particle size range influences image resolution, as well. Our data suggests that during size evaluation of 25 μm PS microspheres, MDRS measured 26.23 ± 0.20 μm for a homogeneous sample measurement (25 μm PS microsphere) and 29.82 ± 0.42 μm for a heterogeneous sample measurement (10 μm and 25 μm PS microsphere mixture), showing deviation in size for the particles in mixture. The ETFE particles has a much broader size range than a monodisperse sample, and therefore the image resolution might be impacted, in that low-resolution images were generated from particles at the lower end of the size range. In addition to these factors, the low refractive index of the ETFE likely also contributes to reducing the particle image resolution. However, given that MDRS could capture well-resolved images from the SVP in the range from 19 to 100 μm covering most SVP range, the MDRS system showed adequate capability of image-based particle analysis (Refer to Table 1).

The performance of MDRS in size and shape measurement was evaluated using monodisperse PS microspheres and SU-8 particles. Our data demonstrates that overall, MDRS system provided closer results to optical microscopy results in both size and shape measurements compared to FlowCam system. Even though FlowCam showed slightly better performance in image analysis, MDRS offered closer results to the specifications in most of the measurements. In shape analysis of polydisperse ETFE particles, MDRS rendered closer morphology values to the values obtained from optical microscopy than the values calculated by the FlowCam except for convexity value. Our data demonstrates that MDRS can provide comparable data with FlowCam. For size analysis**,** % particle size distribution was calculated by MDRS and compared with the two most common FIM systems, MFI and FlowCam. MDRS counted fewer particles in the 10–25 μm size range and greater number in the > 25 μm size range than the MFI and FlowCam. As seen in monodisperse particle characterization, the percentage differences among these methods were less than 5%; The difference between MFI and MDRS was less than 5% and the difference between FlowCam and MDRS was less than 2%. This suggests that MDRS could provide comparable size measurement data to currently used FIM systems. As explained in the Result Section, MDRS system was not able to perform full quantitative analysis such as particle concentration quantification. As technology advances MDRS might be able to offer quantitative data but currently, it is not a viable technique for particle concentration quantitation in the current set-up.

As discussed earlier, MDRS enables particle chemical identification using Raman spectroscopy. Using Raman spectroscopy, each particle can be identified, and its chemical correlation score can be calculated based on the similarity of the particle Raman spectrum to a reference spectrum. To evaluate this capability, PS microspheres and ETFE particles were employed to calculate chemical correlation scores. In our experiment, the chemical correlation scores seemed to be directly influenced by Raman peak S/N which were significantly affected by the sample size such as diameter and thickness, because of the laser spot size (3 μm in diameter) of MDRS. If a particle size is smaller than the laser spot size, the S/N decreases in the particle spectrum due to background interference^18^. Supporting this notion, our data indicated that as the diameters of PS microspheres or ETFE particles decreased, the S/N also decreased. For example, higher S/N was observed from 25 μm PS microspheres compared to 10 μm PS microspheres, even though both 25 μm and 10 μm PS microspheres had larger diameters than the laser spot. In addition to particle diameter, particle thickness seemed to influence the S/N. The average chemical correlation score of 2 μm PS microspheres was 0.96 out of 1, meaning distinguishable Raman spectra with high S/N, although the particle diameter was smaller than the laser spot. In contrast, the average correlation score of ETFE particles in the range of 5–10 μm was calculated as 0.65 out of 1 because of relatively low S/N even with the larger diameters. PS microspheres are spherical, and therefore their thickness and diameters are always same. Since ETFE particles have relatively thin and flat morphological feature compared to the microspheres, their thickness might be much smaller than their diameters. For this reason, the average chemical correlation score of ETFE particles in the range of 5–10 μm was much lower than that of 2 μm PS microspheres.

Next, in the evaluation of the particles formed in the stressed mAb samples, MDRS particle analysis method was applied to characterize the proteinaceous particles providing information on the particle size, shape, size distribution, and chemical identity. Our data reported that different mAbs generated proteinaceous particles having different sizes and shapes, even though they were subjected to the same stress. The size of proteinaceous SVPs formed in Vectibix sample were smaller than those formed in Rituxan sample. Vectibix proteinaceous particles present in the smaller subvisible size range (< 40 μm) and Rituxan proteinaceous particles were distributed across the entire subvisible size range. In terms of particle morphology, proteinaceous particles with different morphology were formed, despite exposure to the same stress. Moreover, their morphology parameters changed in a different manner as the particle size grew. The average morphology values of Vectibix proteinaceous particles remained largely unchanged demonstrating the particle shapes were unaffected by particle growth. In contrast to Vectibix proteinaceous particles, the average aspect ratio, circularity, and convexity values of Rituxan proteinaceous particles decreased and the average elongation value increased as particle growth. This particle morphology change demonstrates that Rituxan proteinaceous particles form more elongated shapes as particle growth.

In chemical identification of proteinaceous particles, the average chemical correlation scores were calculated as 0.35 out of 1 for Vectibix and 0.66 out of 1 for Rituxan with respect to protein aggregate reference spectrum. These scores are lower than the average score of ETFE particles having the same or smaller diameters because of lower S/N. Even though proteinaceous particles ≥ 25 μm were measured to acquire the Raman spectra, these particles showed very low S/N. As discussed in the chemical identification of PS microspheres and ETFE particles, particle thickness is one of the most significant factors directly affecting Raman peak S/N. Therefore, proteinaceous particles might be thinner than ETFE particles in 3D structure, despite the same or larger diameter in 2D structure. Given that Raman spectra of proteinaceous particles were obtained in aqueous solution states, the hydrated particle states could be another reason for low S/N. Though particle identification was successfully achieved from Rituxan proteinaceous particles, further studies are required to improve the Raman signal coming from proteinaceous particles. To identify types of particles, having a large collection or database of Raman spectra would streamline the process of particle identification. Our data also demonstrated that different types of TPs behave differently even under the same stress condition. Therefore, when using MDRS for SVP assessment of TPs, the method should be validated for that specific drug product.

Particle morphology and transparency of the three different reference materials such as PS microspheres, SU-8, and ETFE were compared with those of proteinaceous particles. Among these particles, the ETFE candidate reference material showed the closest physical properties to those of proteinaceous particles. Currently, PS microspheres have been used as particle standards in industry for SVP analysis, but they have different shapes and optical properties from protein aggregates as seen in our experiments. Considering the similarity in morphology and transparency between ETFE and proteinaceous particles, ETFE particles would be more advantageous to reduce the gap between the traditional standard and proteinaceous particle in SVP analysis for TPs quality control, improving the accuracy of the current analysis methods.

## Conclusion

It is important to develop sensitive and selective analytical methods to characterize TPs and their impurities. Our study introduced an analytical method using MDRS to monitor SVPs in TPs and this method was applied to the characterization of proteinaceous particles formed in the stressed mAb drug products. The goal of this study was to expand TPs SVP analysis methods for product quality characterization which impacts public health by introducing a new analytical method. MDRS offered comparable particle morphology and size distribution data to the current orthogonal method, FIM in the evaluation of monodisperse particles (PS microspheres and SU-8) and polydisperse particles (ETFE) which showed the best resemblance to the proteinaceous particles. Our results highlighted the chemical identification capability of MDRS in SVP analysis. In the characterization of proteinaceous particles, MDRS was able to chemically identify individual particles using Raman spectroscopy by calculating their chemical correlation scores based on the similarity of their Raman spectra to the reference spectra along with morphology analysis. Although weak Raman S/N of proteinaceous particles remains a challenge, our study suggests that MDRS may improve and expand the current SVP analysis system and product quality assessment showing its feasibility as an additional SVP analysis tool.

## Methods

### Sample preparation

ETFE particles were produced as described by Ripple et al.^30^ Briefly, ETFE tubing (Saint Gobain, New Jersey) was abraded against a nickel-bonded diamond abrasive disc using a custom-designed abrasion apparatus built at NIST. The abraded particles were washed off the disc with a 0.01% (w/v) Triton X-100 and 0.02% (w/v) sodium azide solution and transferred to a collection vial. The vial was shaken vigorously for 20 s to disentangle the particles. Once the foam dissipated, the particle suspension was filtered through a 53 µm nylon screen to filter out particles larger than 50 µm. After settling the suspension for 8 min, the bottom 10 mL, which was enriched in larger particles, was pipetted out and stored in another vial for final use.

SU-8 photolithographic particles were produced as described by Telikepalli et al.^17^ These particles were fabricated as irregular, monodisperse, two dimensional shapes on a silicon wafer and released into solution after dissolution of a bond layer. The production and release of these particles was performed in a class 100 cleanroom. After the release of the particles, an extensive cleaning procedure was employed to eliminate residual solvents and purify the particles.

Polystyrene microsphere standards were purchased from Duke Scientific through Thermo Fisher Scientific. Monoclonal antibody (mAb) drug products panitumumab (Vectibix®, expiry data: 12/2024, Lot number: 1144255) and rituximab (Rituxan, expiry date: 08/2024, Lot number: 3491321) were obtained from commercial sources. 5 mL of each mAb solution was aliquoted in glass scintillation vial and exposed to a combined (thermo-mechanical) stress condition for 1 h. The aliquoted samples were kept in BenchTop Incubator-Shaker at 70 °C and 300 rpm. The stressed mAb samples were stored at 4 °C until measurement.

DP formulation information can be found below:

Vectibix (panitumumab) 20 mg/mL; Each 5 mL single-dose vial consists of 100 mg panitumumab, 29 mg sodium chloride, 34 mg sodium acetate in water for injection.

Rituxan (rituximab) 10 mg/mL; Each 10 mL single-dose vial contains 100 mg rituximab, polysorbate 80 (0.7 mg), sodium chloride (9 mg), sodium citrate dihydrate (7.35 mg) and water for injection.

### Optical microscopy

Nine SU-8 particles were imaged with a 20X/0.4 numerical aperture objective with Kohler brightfield illumination at 530 nm wavelength on a Leica DMR microscope with an Andor Zyla 5.5 camera. A calibrated stage micrometer was used to find the image pixel size. The particle edge was determined in ImageJ 1.53q using a gray level threshold set at the mean of the image background intensity and the dark center of the particle borders. This procedure matches the recommendation in ISO 13322-1. Particle size was described as equivalent circular diameter (ECD) in microns.

### Morphologically-driven Raman spectroscopy (MDRS)

A Morphologi 4-ID instrument (Malvern Panalytical Ltd), which is a static automated imaging system combined with Raman spectroscopy, was used to evaluate size, shape, counts, transparency, and chemical identification of particles. After resuspension, 20 µl of each sample was loaded onto a quartz microscope slide and covered with a circular-shape quartz coverslip (22 mm in diameter) to form a thin layer. To prevent evaporation, nail polish was applied along the edge of the coverslip. The sealed sample was left resting for 30 min at room temperature to allow particles to settle to the bottom face before measurement. The image detector has a pixel size of 1.25 × 1.25 μm with 5 different magnifications (2.5×, 5×, 10×, 20×, and 50×). The Raman spectrum of each particle was obtained at 50× magnification using a coupled Kaiser optical systems RamanRxn1 spectrometer (a 785 nm semiconductor laser with a power of < 500 mW, and a 3 μm spot size). A 30 s exposure time on a low laser power was used to avoid fluorescence. A chemical correlation score between a particle of interest and a reference was calculated by Morphologi Software. For protein aggregate reference spectrum, 20 proteinaceous particle candidates were selected from a stressed Rituxan sample (70 °C and 300 rpm, 24 h), and their Raman spectra were acquired. Among all the spectra, the one showing the greatest intensities at 1000 cm^−1^ for phenylalanine, at 1230 cm^−1^ for amide III bonds, at 1400 cm^−1^ for asymmetric CH_2_, and at 1670–1690 cm^−1^ for amide I bonds was chosen for the protein aggregate reference spectrum. The chemical correlation scores of protein aggregates were calculated based on the reference spectrum. Particle morphology was characterized using several parameters such as aspect ratio, circularity, convexity, and elongation and a value between 0 and 1 was assigned to each particle Aspect ratio is the ratio of the width (the Feret’s minimum length) to the height (the Feret’s maximum length). Circularity is circumference of equivalent area circle by the actual perimeter of the particle. Convexity is convex hull perimeter divided by actual particle perimeter. Elongation is calculated as 1—aspect ratio. Particle size was described as diameter (ECD) in microns.

### Flow imaging microscopy (FIM)

FlowCam was used as FIM method in all the experiments. For size distribution comparison, both FlowCam and MFI were used together in comparison with MDRS.

### FlowCam

A FlowCam 8400 (Fluid Imaging Technologies, Inc.) flow imaging microscope equipped with a multi-use flow cell (80 × 700 µm, depth x width) and a 10× magnification lens was used for this study. The instrument performance was verified by particle count and size measurement of a 10 µm polystyrene bead standard. In each run, 400 µl of a sample was loaded and analyzed at a flow rate of 0.1 ml/min, followed by a washing step using 10% PCC-54 detergent and 18.2 MΩ·cm deionized (DI) water at a flow rate of 1 ml/min. Auto-image rate and segmentation threshold (dark/light) were set for 15 frames/sec, and 12/12, respectively. Sampling efficiency was calculated as approximately 57.7%. In this experiment setting, less than 200 particles > 2 µm in size and no particles > 10 µm in size were detected from 1 ml of deionized water. The number of particles were counted, and the particle images were obtained from particles in the range of 1–80 µm using VisualSpreadSheet software. Samples were measured in triplicate to confirm the results. Particle size was described as diameter (ECD) in microns.

### Micro-flow imaging (MFI)

A Micro-Flow Imaging DPA4200 flow imaging microscope (Biotechne, Minneapolis, MN) with a set point 3, 4X objective, and 100 µm thick flow cell was used for this study. Neptune 1 mL barrier pipette tips (San Diego, CA) were used to load the sample. Since the ETFE particles rapidly sediment, a previously described method to account for the rapid settling by Ripple et al.^28^ was used to analyze the samples. Between each run, the tubing and flow cell were cleaned using deionized ultrafiltered water and 0.01% Triton X-100, 0.02% sodium azide solution. Particle size was described as diameter (ECD) in microns.

### Supplementary Information

Supplementary Figures.

## Data Availability

The datasets generated during and/or analysed during the current study are available from the corresponding author on reasonable request.
