# Supplementary material for: Morphologically-Directed Raman Spectroscopy as an Analytical Method for Subvisible Particle Characterization in Therapeutic Protein Product Quality
Source: Sci Rep. 2023 Nov 22;13:20473. doi: 10.1038/s41598-023-45720-0 (PMC10665318; doi:10.1038/s41598-023-45720-0)
Supplement: Supplementary file 1 — Supplementary Figures. [file 41598_2023_45720_MOESM1_ESM.docx]

**Analytical Method for Subvisible Particle Characterization in Protein Therapeutics Product Quality**

Minkyung Kim^a^, Youlong Ma^b^, Charudharshini Srinivasan^b^, Thomas O'Connor^b^, Srivalli N. Telikepalli^c^, Dean C. Ripple^c^, Scott Lute^a^*, and Ashwinkumar Bhirde^a^*

^a^ Division of Biotechnology Research and Review II, Office of Biotechnology Products, Office of Pharmaceutical Quality, Center for Drug Evaluation and Research, U.S. Food and Drug Administration, Silver Spring, MD, USA

^b^ Division of Product Quality Research, Office of Testing and Research, Office of Pharmaceutical Quality, Center for Drug Evaluation Research, U.S. Food and Drug Administration, Silver Spring, MD, USA

^c^ Biomolecular Measurement Division, National Institute of Standards and Technology, Gaithersburg, MD, USA

**Supporting Material:**

*
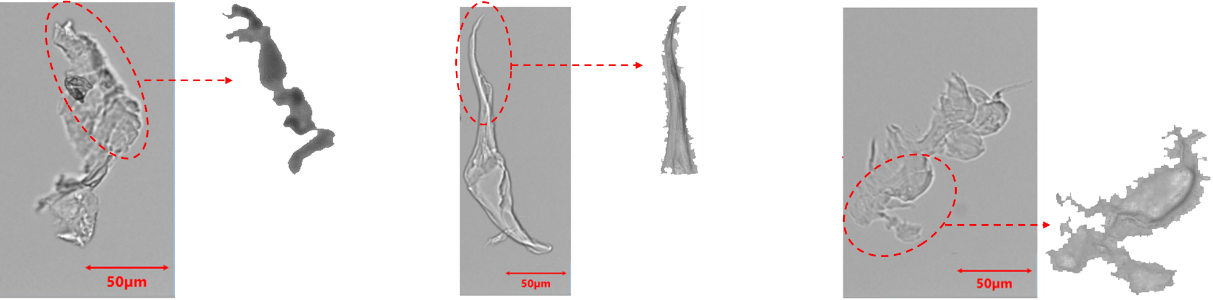
*

**Figure S1.** Examples of image fragmentation of ETFE particles. The double image regions, where the image fragmentation was observed, were circled in red. The MDRS system recognized these regions as different particles and produced new particle images. The particles of interest were presented under microscope and compared with their captured images in the measurement.

**MDRS:** 10.91 ± 0.29, 29.82 ± 0.42 μm

**FIM:** 10.87 ± 1.13, 27.91 ± 1.41 μm

**MDRS:** 26.23 ± 0.20 μm

**FIM:** 27.70 ± 2.78 μm

**MDRS:** 10.81 ± 0.21 μm

**FIM:** 10.81 ± 2.26 μm

**A. B. C.**

**Normalized Particle Counts**

**Diameter (μm)**

**Figure S2.** Size measurement analysis of PS microspheres standards using MDRS and FIM. The 10 μm (**A**) and 25 μm (**B**) microsphere standards were measured separately and, in a mixture (**C**). Particle sizes are obtained from 3 separate measurements (n = 3) reported as the mean ± standard deviation. Segmentation threshold (dark/light) were set for 20/20 in the FIM measurements.


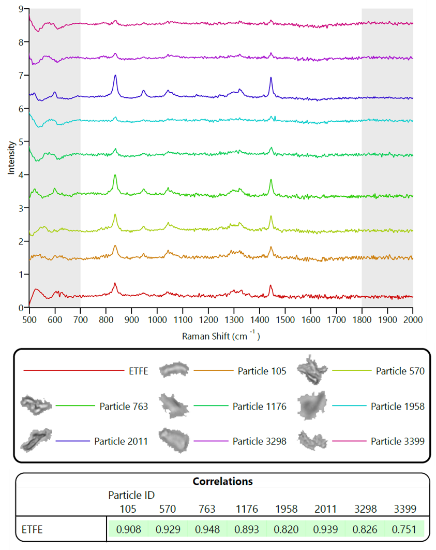

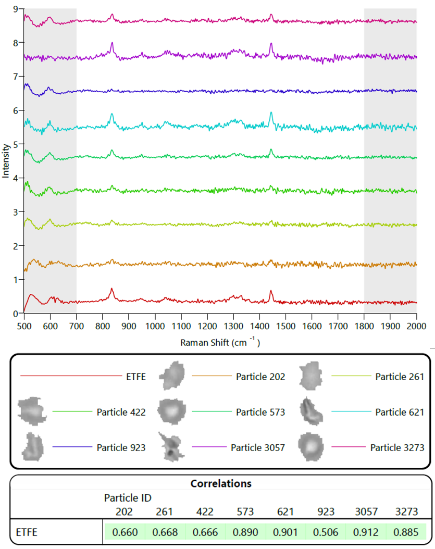

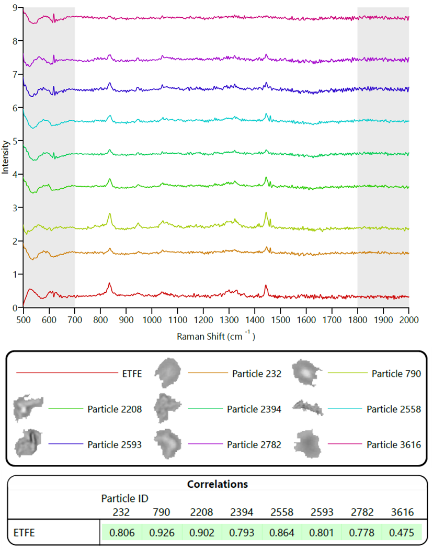


**D. E.**

**A. B. C.**


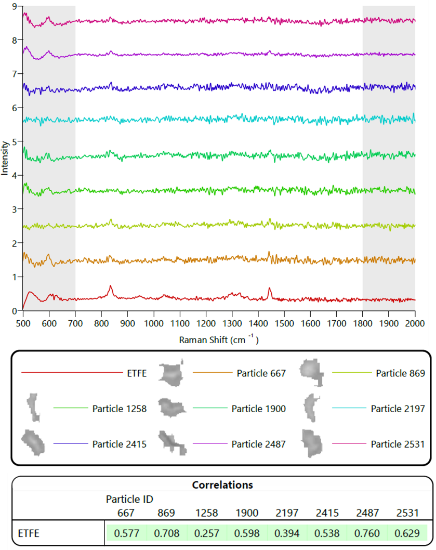

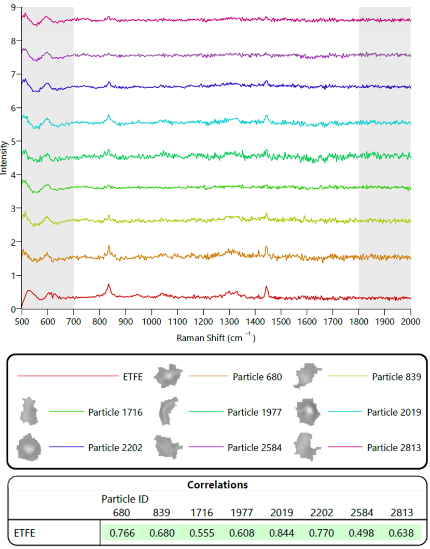


**Figure S3.** **Chemical identification of ETFE particles in 5X E5S82A reference material.** Representative Raman spectra of ETFE particles in size of **(A)** 25 > CE Diameter ≥ 15, **(B)** 15 > CE Diameter ≥ 10, **(C)** 10 > CE Diameter ≥ 8, **(D)** 8 > CE Diameter ≥ 6, and **(E)** 6 > CE Diameter ≥ 5. The region of 500 - 2000 cm^-1^ in each spectrum was compared with ETFE reference spectrum (red) and their correlation scores were calculated based on the peak similarity. Characteristic peaks found in an ETFE polymer were highlighted with gray dashed lines at 835 cm^-1^, and 1444 cm^-1^.

**Diameter (μm)**

**Chemical Correlation Score**

**Figure S4.** Scattergram displaying the association between chemical correlation score and particle size. The chemical correlation scores of Rituxan proteinaceous particles (>20 μm) with respect to protein aggregate were used to plot this scattergram.

**164.32 ± 4.41**

**135.42 ± 6.39**

**156.10 ± 6.86**

**58.97 ± 8.15**

**143.45** **± 3.87**

**Figure S5.** The average mean intensity values of PS microspheres (orange), SU-8 particles (green), ETFE particles (blue), and proteinaceous particles formed in the stressed Rituxan (yellow) and Vectibix (gray) samples.
